# Robust and highly performant ring detection algorithm for 3d particle tracking using 2d microscope imaging

**DOI:** 10.1038/srep13584

**Published:** 2015-09-02

**Authors:** Eldad Afik

**Affiliations:** 1 Department of Physics of Complex Systems, Weizmann Institute of Science, Rehovot 76100, Israel

## Abstract

Three-dimensional particle tracking is an essential tool in studying dynamics under the microscope, namely, fluid dynamics in microfluidic devices, bacteria taxis, cellular trafficking. The 3d position can be determined using 2d imaging alone by measuring the diffraction rings generated by an out-of-focus fluorescent particle, imaged on a single camera. Here I present a ring detection algorithm exhibiting a high detection rate, which is robust to the challenges arising from ring occlusion, inclusions and overlaps, and allows resolving particles even when near to each other. It is capable of real time analysis thanks to its high performance and low memory footprint. The proposed algorithm, an offspring of the circle Hough transform, addresses the need to efficiently trace the trajectories of many particles concurrently, when their number in not necessarily fixed, by solving a classification problem, and overcomes the challenges of finding local maxima in the complex parameter space which results from ring clusters and noise. Several algorithmic concepts introduced here can be advantageous in other cases, particularly when dealing with noisy and sparse data. The implementation is based on open-source and cross-platform software packages only, making it easy to distribute and modify. It is implemented in a microfluidic experiment allowing real-time multi-particle tracking at 70 Hz, achieving a detection rate which exceeds 94% and only 1% false-detection.

The study of dynamics often relies on tracking objects under the microscope. Indeed, precise and robust particle tracking is essential in many fields, including studies of micro-Rheology^1^,^2^, chaotic dissipative flows^3^,^4^, feedback for micro-manipulation^5^, and other soft condensed matter physics and engineering problems. Moreover, microfluidic systems play a growing role as part of lab-on-a-chip apparatus in micro-chemistry^6^,^7^, bioanalytics^8^,^9^, and other bio-medical research and engineering applications^10^. Yet, detailed characterisation of the flow and transport phenomena at the micro-scale is still a non-trivial task. In general, the motion is three dimensional and automated tracking is cumbersome from the perspective of both instrumentation and software. Setting up several viewing angles as done for large systems^11^ becomes even more complicated in microscopic systems, while scanning through the third axis, e.g. confocal microscopy, clearly compromises temporal resolution and concurrency. In this work the three-dimensional positions of fluorescent particles are inferred from the information encoded in the diffraction rings which result from out-of-focus imaging, converting the 3d localisation problem to an image analysis problem of ring detection.

The development of the method presented here was motivated by the study of pair dispersion in a chaotic flow^12^, taking place in a microfluidic tube of 140 μm—the observation volume is larger by more than three orders of magnitude with respect to those reported in Refs. 5 and 13. The experiments consist of tracking tracers advected by the flow, at seeding levels of several tens to hundreds in the observation window, where it is necessary that the particles are resolved even when nearby to each other. The typical flow rates dictate sampling rates of 70 Hz whereas the statistical nature of the problem requires data acquisition over weeks. Using a standard epi-fluorescence microscope and the fact that the parameters of the most visible ring can be mapped to the 3d position of the tracer, the particle localisation problem is converted to a circle detection problem. The constraints set by the nature of the experiment require an image analysis algorithm that is robust not only to the noise of the image acquisition process, but to rings overlaps, inclusions and occlusions as well. The typical complexity of the images is exemplified in a sub-frame from our experiment presented in Fig. 1a. In addition, the data flow is of about 180 GB/h, an overwhelming rate which demands the optimisation of the algorithm for real-time analysis.

In this presentation I will focus on the development of an algorithm for this purpose. The key steps for achieving high-performance are introduced following the presentation of the main concepts which contribute to the robustness of the algorithm. The application for particle tracking is presented and discussed in the Supplementary Information; further technical details of the optical apparatus can be found in the Methods section.

Imagine for the moment that you have successfully identified which of the pixels in the image reside on a ring. The issue of doing so will be addressed later on. Given this set of coordinates, it may seem straightforward to find the parameters of the circles which best fit them. However there is a missing piece of information here, that is, which sub-sets of pixels belong together to form a ring. Moreover, we do not know a priori how many rings there are in the image and there may be false detected coordinates which we would like to disregard. Therefore, we need some method to classify/cluster the coordinates into sub-sets, each sub-set matching a single ring.

A circle in a two-dimensional image is uniquely specified by three parameters. In this work two designate the centre of the circle and the third specifies its radius. The parameter space of all possible circles is therefore three-dimensional. One can detect circles in an image by mapping the image intensity field to the circle parameter space. Peaks in this parameter space imply a circle well represented in the image. One approach to achieve this mapping is via a discrete Radon transform, which for the purposes of this presentation translates to convolving the image with a mask of a ring^14^. Since each candidate radius calls for a separate convolution, this results in visiting all the pixels in the image over and over. Recall that the outer-most ring is sufficient for 3d localisation of the imaged particles. Hence it is worth noting that even at moderate rings densities, the pixels lying on the outer-most ones consist a small fraction of the pixel population, less than 2% in my case. When there are more than a couple of potential radii this procedure would perform a plethora of useless computations^14^. This fact directs to another approach, which may seem equivalent yet explicitly exploits this information sparsity—the circle Hough transform^15^: each pixel votes for all the candidate points in the parameter space of which it may be part. In this way, every pixel is visited once, potentially reducing the computation time by orders of magnitude. The discrete version of the parameter space is commonly referred to as the array of accumulators. During the voting procedure each vote increments an accumulator by one. Alas, in the literature of Computer Vision and Pattern Recognition it is well known that the standard circle Hough transform is rather demanding both for large memory requirements, which grow with the radii range, as well as for its 3d nature which renders peak finding in the parameter space a difficult task to tackle^16^,^17^.

One way to address these computational challenges is to resort to lower dimensionality circle Hough transforms, but these usually miss circles having nearby centres and are less robust, resulting in higher false positive and false negative errors rates^16^. Another path is to randomly sub-sample the information content in the image, giving way to non-deterministic methods; see Ref. 17 and references therein. However, due to their random nature these methods suffer from inferior detection rates and accuracy when compared with the deterministic ones^17^.

For these reasons I developed an algorithm which is an offspring of the full 3d-circle Hough transform, yet the local maxima detection issues are addressed and it shows high performance and a small memory footprint.

## Results

**Figure 2:**
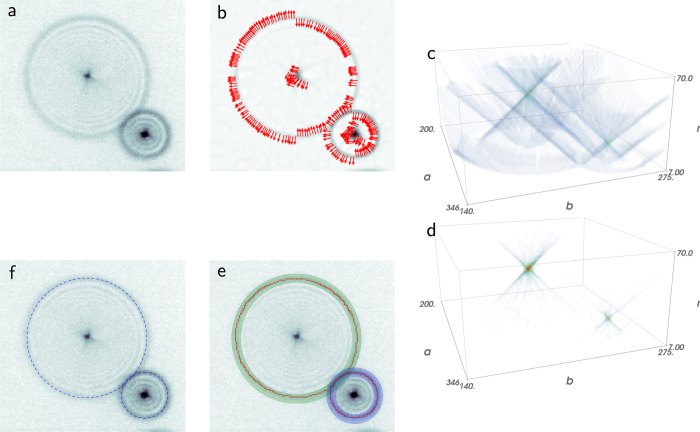
Algorithm outline. (**a**) raw sub-image containing two fluorescent particles; note that the inner rings of each particle are thinner than the outer most one. This scale separation admits suppression of all but the outer most ring via Gaussian smoothing (to ease visualisation the contrast was enhanced in the images on the expense of the central peak of the diffraction pattern); (**b**) ridge detection: the ridges are defined using a differential geometric descriptor and shown here as arrows representing *X*^–^, the principal direction, corresponding to *k*^–^, the least principal curvature, which is plotted in the background. The arrows originate from the ridge pixel. Note that the inner rings are successfully suppressed based on the scale separation. To ease visualisation every second detected ridge is omitted; (**c**) circle Hough transform: directed ridges → circle parameter space; (**d**) local maxima detection: radius dependent smoothing of the parameter space as well as normalisation by 1/r and thresholding greatly emphasise the local maxima representing the rings in the image; (**e**) sub-pixel accuracy: based on the detected rings, annulus masks (blue and green annuli in the figure) allow classification of ridge pixels (red points) and sub-pixel accuracy is achieved via circle fitting. Note the discarded directed ridges of the central peak (in (**b**)) as they do not belong to any local maxima in the processed circle parameter space (**d**); (**f**) the output: best fit circle for the ridge pixels of the outer-most ring of each particle.

The key steps of the algorithm are conceptually outlined as follows: (i) detect directed ridges; (ii) map the directed ridges to the parameter space of circles; (iii) detect local maxima via radius dependent smoothing and normalisation; (iv) classify the coordinates of the ridge pixels according to the peaks in the circle parameter space, and fit each sub-set to a circle, achieving sub-pixel accuracy. This outline is presented graphically in Fig. 2 for a small sub-frame containing two fluorescent particles.

### Directed ridge detection & votes collection

The first step is locating the pixels of interest. Like many other feature detection algorithms, the standard circle Hough transform relies on an edge detection step, where edges are the borders of dark and bright regions. As the images contain rings rather then filled circles, I chose to implement an algorithm that detects ridges, thin curves which are brighter than their neighbourhood, rather than edges. This exhibits better consistency. First note that the ring of interest is thicker than the inner ones. This is advantageous as the image admits scale selection^18^—the inner rings can be suppressed using a Gaussian smoothing having the appropriate scale, approximately that of the most visible ring. Ridges are then found using a differential geometric descriptor^18^, which defines ridge pixels using the following two properties: (i) Negative least principal curvature, *k*^–^ < 0; (ii) *k*^–^ is a local minimum along the direction of the associated principal direction *X*^–^. The principal curvatures are the eigenvalues of the Hessian, the matrix of the image second spatial derivatives. Here *k*^–^ and *X*^–^ denote the smaller principal curvature and the corresponding eigenvector. This is demonstrated for the two fluorescent particles in Fig. 2a, where this sub-image is analysed for directed ridges, represented by arrows overlaid on the *k*^–^ field as background of Fig. 2b. Note that *X*^–^ is collinear with the direction to the centre of the ring. I use this to significantly reduce the complexity of the voting procedure, in a similar way to the gradient directed circle Hough transform^19^—each directed ridge pixel votes for all candidate points in the 3d parameter space of circles, provided that the circle centre is within the *r*_*min*_ to *r*_*max*_ range, directed along X^–^.

### Local maxima detection in a noisy parameter space: radius dependent smoothing & normalisation

As votes from the ridge pixels accumulate, each ring in the image transforms into two mirroring coaxial cones, aligned along the radius axis, having a joint apex. This procedure results in a discrete scalar function over a 3d box. This is demonstrated in Fig. 2c. The coordinates of the apexes, which are the local maxima of this function, are the candidate circle parameters. In practice, there are many sources which render the resulting circle parameter space very noisy: The raw image is a discrete representation of the intensity field, the image acquisition process itself is not noiseless, and the image complexity mentioned above, all may result in errors in the detected ridge position and direction, as well as false ridge detection and false negatives. Note that the deviation from ideal voting due to the error in determining the ridge direction grows linearly with the radius. Therefore, each equi-radius level of the ring parameter space is smoothed using Gaussian weights, whose width is proportional to the radius. Next, note that finding local maxima in the 3d parameter space requires the comparison of accumulators in different equi-radius levels, which asks for some normalisation as larger rings are expected to receive more votes. This leads to a natural normalisation by 1/*r*, following which an ideal ring is expected to receive 2*π* votes. Figure 2d shows how this procedure simplifies the parameter space. Local maxima can then be located by nearest-neighbours comparison.

### Ridge points classification & sub-pixel accuracy

The local maxima identified in the parameter space induce a classification on the ridge coordinates: for each peak an annulus mask is formed and a best fitting circle is found for all ridge coordinates within the annulus. Ridge pixels which are not covered by any annulus mask are not fitted for. This is desired as these usually result from non-circular features in the image or noise. See the example in Fig. 2e. In this way sub-pixel precision is achieved.

### Empirical detection and error rates

Examining the robustness of the algorithm on the experimental data reveals a detection rate that exceeds 94% with only 1% false-detection; for further details see Robustness assessment in the Methods section.

To demonstrate the excellence of these results let us compare the robustness with a recently published algorithm—the EDCircles algorithm introduced in Ref. 20. Founded on the mathematical theory of perception^21^ it detects contiguous edge segments and employs the Helmholtz principle for controlling false detections. It was chosen as the competitor for this examination for two main reasons: (i) it is parameter-free and so the comparison is insensitive to the choice of input parameters; and (ii) the results and the results presented in the manuscript^20^ are very promising—the EDCircles was demonstrated to exhibit a much better detection rate when compared with the state-of-the-art lower dimensionality Circle Hough Transform implemented in OpenCV^22^, referred to as 21HT in Ref. 16.

In practice, the EDCircles showed a detection rate lower than 61% and nearly 2% false-detection. While the EDCircles detected 21% of the rings missed by the algorithm proposed here, the latter detected successfully more than 88% of those missed by its competitor for this comparison; examples can be found in Supplementary Fig. S3; further details of the test and results are summarised in Comparative assessment of the algorithm robustness in the Methods section.

For precision and accuracy estimation, in particular in the light of particle localisation, see Experimental details and Precision assessment in the Methods section as well as Supplementary Fig. S1 and the accompanying caption.

### Key algorithmic optimisations for memory requirement & temporal performance

As was mentioned above, it is desired for our purposes to have the images processed in real-time. Here I briefly outline the key ideas behind the optimisation of the algorithm, the full details are available in the open-source code itself (see the Methods section). The first key point for the algorithm optimisation is the splitting of the voting procedure—the votes are recorded for each ridge pixel as it is detected, such that ridge detection and votes collection are done in *one-pass*.

The population of the parameter space is performed at a separate stage, which leads to the second key point. Instead of holding an array for the full parameter space, only two sub-spaces are maintained, consisting of three consecutive equi-radius levels; the first for the raw parameter sub-space, the second for the smoothed and normalised one, where local maxima are searched for. The equi-radius levels are populated and processed one by one. Only the accumulators exceeding an integer vote threshold are regarded as hotspots and are mapped to the smoothed and normalised sub-space. Each time a radius-level is completed, regarded here as the “top” one, the hotspots in the level beneath, the “middle” one, are verified to exceed a pre-set floating point threshold, a fraction of 2*π*. Those which do are searched for local maxima by a nearest neighbour comparison within a 3 × 3 × 3 voxels box. Once this search is completed, the “bottom” level is no longer needed and a cyclic permutation takes place where the “bottom” level becomes the new “top”. This allows *memory recycling* and avoids the need to initialise big arrays of zeros to represent the full 3d parameter space.

*Registering modified array elements and undoing* is the last key point. The radius-levels are required to be blank prior to their population. Recalling the sparsity of the parameter space (see Fig. 2c,d for example), going over all its elements is a waste of processing time. Instead, each time an array element is modified for the first time its indices are registered. Once the search for peaks is done, all modifications to the “bottom” radius-level are undone, preparing it for reuse. This lifts the need to clean the whole array.

The combination of *memory recycling* with *registering modified array elements and undoing* reduces the computation time and in my case results in a nearly ten times less memory consumption. In fact, the size of the arrays representing the parameter space kept in memory is now fixed and no longer grows with the radii range. For preliminary testing purposes, I first implemented the algorithm outlined in the beginning of the Results section (as well as in Fig. 2) using convolutions and other array based operations. The final implementation, inspired by the circle Hough transform and including the above optimisations, is more than 50 times faster. This is attributable to the reduction in the number of operations required once the sparsity of the data is taken advantage of. Further details and explanations can be found in the Methods section and the Detailed algorithm section of the Supplementary Information.

## Discussion

In this work I have presented a new algorithm to analyse images of complex annular patterns. Image complexity and noise often result in a challenging parameter space where local maxima are difficult to find, a problem not addressed within the classical Hough transform algorithm. The main novelty introduced here to overcome this difficult task and to gain robustness are the radius dependent smoothing and normalisation. The resulting detection and error rates are very promising, even more so in the light of alternative methods. As it was already mentioned in the introduction the non-deterministic or randomised methods typically provide a gain in the temporal performance but suffer in reliability when it comes to detection and error rates^17^. The EDCircles algorithm^20^ was chosen as a competitor for the comparative assessment of robustness mainly as it was reported to outperform the state-of-the-art implementation of the natural competitor—OpenCV’s deterministic Circle Hough Transform^22^. The algorithm proposed here demonstrated a detection rate higher by more than 50% and a nearly three times smaller false reports rate.

Several algorithmic concepts have been introduced to improve memory requirements and temporal performance, of highest importance are those referred above as *registering modified array elements and undoing* as well as *memory recycling*. These have been empirically shown to reduce memory consumption by nearly ten times and result in an over fifty times faster analysis rate. These can be advantageous for other algorithms as well, particularly when the data is sparse.

Though the development of this algorithm was motivated by the analysis of fluorescence microscopy images, it is more general and can be applied to other cases as well. The interpretation of the Hough transform as a classification/clustering algorithm has a wider potential than merely image analysis. To name a physical example is the case of particle jets emerging from several sources of unknown loci. A dataset consisting of the positions and momenta of the particles at a certain time is analogous to that of the directed ridges and the jet sources can be identified with the local maxima over the parameter space.

The method I introduced above is currently implemented in an experiment which requires long unsupervised measurements lasting for days at high temporal resolution, sampling a volume of interest which contains tens to hundreds of particles. Thanks to the fact that the whole volume of interest is sampled at once by a single 2d image, concurrency is achieved. The use of LED and the relatively short exposure times can be potentially exploited to avoid photo-damage and bleaching. It is demonstrated to be robust to the overlap, inclusion and occlusion of the ring pattern of the imaged particles. It features high performance admitting real-time applications. A discussion of this approach for particle tracking in light of other methods [34,35,36,37,38] can be found in the second section of the Supplementary Information.

This method paves the way for studies of 3d flows in microfluidic devices. Its robustness to the vicinity of particles to each other allows to study the dynamics of particle pairs^12^, triples, etc. As such it also has a potential for biomedical research. A possible immediate application is the detailed characterisation of the transport induced by the presence of cells in confined flows, a phenomenon presented in Ref. 23. Together with the development of high signal tracers, labelling techniques and sensitive cameras, this method may be useful in other life sciences studies such as cellular trafficking^24^, cell migration^25^ and bacterial taxis^26^.

## Methods

### Algorithm implementation

I implemented this algorithm relying on freely available open-source and cross-platform software packages only. The source is available online (https://github.com/eldad-a/ridge-directed-ring-detector). Most of the heavy lifting is achieved using the Cython language^27^. It has a Python-like syntax from which a C code is automatically generated and compiled. This allows the code to be short and easy to read while enjoying the performance of C. For example, this implementation exploits the Numpy/Cython strided direct data access^27^,^28^ by fully sorting the votes. In the image pre-processing step the image is smoothed using a Gaussian convolution and the smoothed image spatial derivatives are calculated using a 5 × 5 2^nd^ order Sobel operator; these operations are done using OpenCV’s Python bindings^22^.

The equi-radius levels of the circle parameter space are smoothed using Gaussian weights 
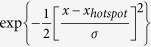
, whose width is proportional to the radius σ(*r*) ∝ *r*. The explicit form, σ(*r*) = 0.05*r* + 0.25, was found empirically. The slope coefficient is interpreted as accounting for ~0.1 rad uncertainty in the direction of *X*^–^.

### Experimental details

The imaging system consists of an inverted fluorescence microscope (IMT-2, Olympus), mounted with a Plan-Apochromat 20 × /0.8NA objective (Carl Zeiss) and a fluorescence filter cube; a Royal-Blue LED (Luxeonstar) served for the fluorophore excitation. A CCD (GX1920, Allied Vision Technologies) was mounted via zoom and 0.1× c-mount adapters (Vario-Orthomate 543513 and 543431, Leitz), sampling at 70 Hz, 968px × 728px, covering 810 μm × 610 μm laterally.

The experiments were conducted in a microfluidic device, implemented in polydimethylsiloxane elastomer by soft lithography, consisting of a curvilinear tube (see grey broken line in Supplementary Fig. S2a). The rectangular cross-section of the tube was measured to be of 140 μm depth and 185 μm width. The working fluid consisted of polyacrylamide in aqueous sugar (sucrose and sorbitol) syrup, seeded with fluorescent particles (1 micron 15702 Fluoresbrite^®^ YG Carboxylate particles, PolySciences Inc.). The flow was driven by gravity.

For the empirical calibration, the same working fluid was sandwiched between two microscope glass slides. The separation distance between the slides was set to 161 μm by micro-spheres (4316A PS NIST certified calibration and traceability, Duke Standards) serving as spacers. The microscope objective was translated in steps of 2 μm to acquire images of the tracers at different off-focus distances Δ*z*. The microscope focus knob was manipulated by a computer controlling a stepper motor. During the rest stages of the objective, the ring radii of every detected tracer were averaged over 210 frames spanning 3 s. Due to the high viscosity of the fluid, 1100 times larger than water viscosity, tracer motion due to diffusion is negligible during this time interval. The median of the estimated standard-deviations of the data presented in Supplementary Fig. S1a is 0.03px and the maximal is 0.27px. In practice, to account for the uncertainties in finding the focal position and due to optical aberrations, 25 tracers dispersed throughout the observation volume were accounted for. Their curves were aligned via shifting Δ*z* by the larger root of a quadratic polynomial fit. Then, the conversion function *r*^−1^(*r*) was obtained by inversion of the quadratic polynomial fit accounting for all the data together; see Supplementary Fig. S1b. The resulting root-mean-squared-error, 
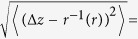
 1.97 μm, and the maximal measured absolute error is 5.35 μm; these reflect the uncertainty due to the empirical calibration procedure taken here. Finally, the out-of-focus distance of the objective Δ*z* has to be converted to the physical distance via multiplication by the ratio of the refractive indices, 1.58 in this case. The observed axial range exceeds 180 μm.

### Robustness assessment

In order to estimate the robustness of the algorithm, images from the chaotic flow experiment were analysed and cropped to a sub-region of 340px × 370px. The analysis results of 600 such sub-frames were examined. These sub-frames contained 14.3 rings on average, out of which 67.7% were in ring clusters (overlap and inclusion configurations). This examination shows an average of 6.8% False-Negative errors. In some sub-frames a ghost ring would appear accompanied by a strong distortion of the tracer image in its real position. This is attributed the microfluidic walls and observed only when a particle is very close to the wall. Excluding from the statistics two such tracers, the False-Negative error rate is reduced, corresponding to a detection rate of 94.7%. This examination does not show any significant sensitivity to rings overlap and inclusion. On the contrary, 95.5% of the rings in clusters were identified correctly while only 0.8% of the reported rings in clusters were non-existing particles.

### Comparative assessment of the algorithm robustness

To demonstrate the high-quality of the above results, a comparison was made against the on-line demo of the EDCircles, provided by the authors of Ref. 20. The tests were conducted on a subset of 151 images taken from the same experiment as in the Robustness assessment above, cropped to the same sub-region of 340px × 370px. In this case the images were first cropped and exported to the PNG format prior to the analysis, the format for which the EDCircles on-line demo exhibited the best detection rate. In this comparison rings whose centre lay outside the cropped image were not considered, as well as those whose visible perimeter was less than a half of the complete one; the average ring number was found to be 13.4. Few examples are presented in Supplementary Fig. S3.

The EDCircles demo detected 60.9% of the rings (False-Negative error rate of 39.2%); in contrast, the method proposed here showed a detection rate of 94.3%. While 1.7% of the reported detections by the EDCircles demo were False-Positive, only 0.6% of the rings reported by the new algorithm presented here were non-existing or erroneous ones. Out of those particles missed by the proposed method 21.1% were detected by the EDCircles demo; in contrast, 88.6% of those missed by the competitor algorithm were detected by the one proposed here.

### Performance assessment

The performance assessment is based the analysis of 1500 full frames containing 50 particles on average; for a typical example see Supplementary Fig. S2a. The test was run on an i7-3820 CPU desktop, running Ubuntu linux operating system. A single process analysed at an average rate of 6.28 Hz. To achieve higher performance as required for our experiments, I use the multiprocessing package of Python, exploiting the multi-core processors available on modern computers. The analysis rate scales linearly with the number of processes. No deterioration of the processing rate per core was noted (tested up to twice the core number with hyper-threading). Based on a producer-consumer model, one can even transparently distribute the workload among several computers if needed. This is partially attributable to the small memory footprint of the algorithm.

### Precision assessment

To estimate the precision of the presented localisation method, smoothing splines were applied to the reconstructed trajectories providing an estimator for the error variance σ^2^; see Ref. 29. The mean values are as follows: σ_*x*_ = 0.134 μm, σ_*y*_ = 0.135 μm and σ_*z*_ = 0.434 μm, for *x* and *y* denoting the lateral coordinates, and *z* the axial one. This axial uncertainty corresponds to 0.3% of the axial range covered by the particles. According to the data provided in Ref. 5 a value of 0.1% was achieved and a similar one in Ref. 13. The uncertainty estimates reported here account for noise which rise not only due to the image analysis and the multi-particle scenario, but also due to other sources, namely the motion of the particles and the linking procedure. The details are as follows.

From a 3 m30 s measurement, 2014 trajectories which span more than 1s were analysed (discarding shorter ones). Particle positions, converted to microns, were linked to reconstruct their trajectories; for a sub-sample see Supplementary Fig. S2b. The linking algorithm was adapted from the code accompanying Ref. 30, generalised to n-dimensions, the kinematic model was modified to account for accelerations, and a memory feature was introduced to account for occasional misses. Finally, a natural cubic smoothing spline was applied to smooth-out noise and obtain an estimate of particle velocity and acceleration^31^,^32^. The smoothing parameter was automatically set using Vapnik’s measure, using a code adapted from the Octave splines package^33^.

## Additional Information

**How to cite this article**: Afik, E. Robust and highly performant ring detection algorithm for 3d particle tracking using 2d microscope imaging. *Sci. Rep.* **5**, 13584; doi: 10.1038/srep13584 (2015).

## Supplementary Material

Supplementary Information

## Figures and Tables

**Figure 1:**
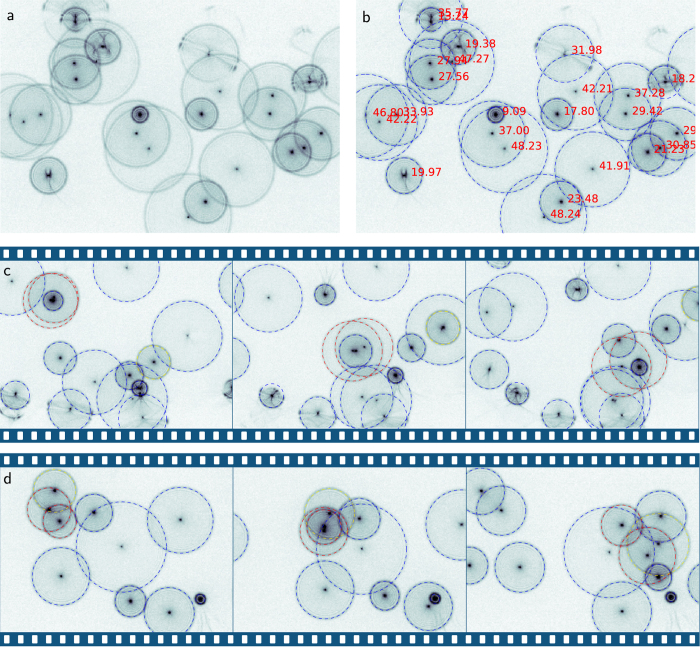
Snapshots from the experiment and a demonstration of the algorithm robustness. (**a**) typical image complexity is exemplified in an unprocessed sub-frame consisting of 1/9 part of the full frame, corresponding to lateral dimension of 215 μm × 315 μm. The axial range available for the particles is 140 μm. (**b**) the corresponding analysis result; in red are the radii in pixels units. (**c**,**d**) time sequences of sub-frames (400 ms each). Red coloured particles in (**c**) demonstrate pair dispersion, in which the algorithm is required to resolve rings with similar parameters. The yellow particle in (**d**) shows radius change corresponding to a downwards translation. Each sub-frame in (**c**,**d**) images a box which lateral dimensions is 190 μm × 270 μm.
