# Supplementary material for: Robust and highly performant ring detection algorithm for 3d particle tracking using 2d microscope imaging
Source: Sci Rep. 2015 Sep 2;5:13584. doi: 10.1038/srep13584 (PMC4557086; doi:10.1038/srep13584)
Supplement: Supplementary Information [file srep13584-s1.pdf]

Supplementary Information for:

Robust and highly performant ring detection algorithm for 3d particle tracking using 2d microscope imaging

Eldad Afik
Department of Physics of Complex Systems,
Weizmann Institute of Science,
Rehovot 76100, Israel

email: eldad.afik@weizmann.ac.il

To keep the main presentation succinct many details of the proposed algorithm and the application for particle tracking under the microscope were left out of the main text. These can be found below. The Supplementary Information opens with a more detailed and technical presentation of the algorithm, followed by the discussion of the application of the proposed algorithm for particle tracking, in particular in the light of the available alternatives; the closing section contains the supporting figures: (i) an empirical calibration curve of the ring radius to the out-of-focus distance is shown in Supplementary Fig. S1; the error bars provide an estimate of the precision of the proposed method resulting from the combination of the algorithm with the optical system together; (ii) Supplementary Fig. S2 shows a sample of 3d particle trajectories reconstructed based on the proposed method; and finally (iii) examples from the comparative assessment of the algorithm robustness referred to in the manuscript and described in the Methods section can be found in Supplementary Fig. S3.

Detailed algorithm

As mentioned in the main text, the standard circle Hough transform is often avoided not only for its challenging local maximum detection in noisy 3d space but for its heavy memory requirements as well. The standard circle Hough transform requires a 3-dimensional array of accumulators. The coordinates of each array element are the parameters of a candidate circle. The value of the accumulator at these coordinates indicates how well this circle is represented in the image. Code optimisation for high-performance and small memory footprint is achieved following this scheme:

1. *Image pre-processing step:* the image is smoothed using a Gaussian convolution and the smoothed image spatial derivatives are calculated using a 5×5 2nd order Sobel operator [22]. Using these derivatives, the local least principal curvature k_{-} is estimated as the smaller eigen-value of the Hessian matrix.

2. *One-pass ridge detection and votes collection:* for each pixel in k_{-} which is smaller than a pre-defined curvature threshold (the latter is no greater than zero), the corresponding X_{-} is calculated. If this pixel is found to be a local minimum along the direction of X_{-}, its coordinates are recorded in the ridge container. At this stage, its votes are collected as well, that is, the potential circles parameters to which it may belong.

3. *Sort the votes stack according to the radii:* this allows performing the parameter space incrementing procedure equi-radius level by level. In order to achieve higher performance,

the votes are further sorted by the row index and then by the column index exploiting the numpy/cython strided direct data access [27, 28]. For this reason each circle parameter triple is represented as an integer using a bijection.

4. *Circle parameter space population and local maximum detection via radius-dependent smoothing and normalisation:* This is done using two arrays representing a sub-space of the full circle parameter space. Each consists of 3 consecutive equi-radius levels; the first for the raw accumulators sub-space, the second for the smoothed and normalised one, where local maxima are to be searched for. There are two votes thresholding steps: an integer threshold for the raw accumulators and a floating point threshold, a fraction of 2π, for the smoothed and normalised array elements. In describing the procedure it is assumed that the $r-2$ and $r-1$ levels have already been populated in both sub-space triples and the r levels are blank, i.e. all zeros. As long as the votes drawn from the votes stack point to the same radius, the corresponding radius-level is populated by incrementing the indicated accumulator. Recall that the votes are fully sorted hence all votes pointing to a certain voxel will come out from the stack in a row. Every time a new circle parameter triple is encountered, its coordinates are recorded as modified. In case the previously incremented voxel has surpassed the 1st votes threshold its coordinates are recorded as a hotspot – a circle candidate. Once there are no more votes for this r-level, it is mapped to the second subspace: for each hotspot voxel a spatial average is calculated, weighted by a Gaussian function, which width is linearly dependent on the radius; the value of the average is then normalised by $1/r$. After mapping all the hostpots of the current r-level, a local maximum is searched for among the hotspots of the $(r-1)$-level which pass the 2nd votes threshold. This is done using a nearest neighbours comparison within a $3\times3\times3$ voxels box. Array elements which are local maximum and exceed the threshold are registered as rings. Once all hotspots have been processed, all modifications to the $(r-2)$-level are undone as its data are no longer needed. By this it is made ready to be regarded as the next r-level and a cyclic permutation among the levels takes place. In practice, this is performed by accessing the equi-radius levels using the modulo operation – the radius indices are calculated using $r \pmod 3$.

5. *Sub-pixeling via circle fit:* the detected ridge coordinates are subjected to a circle fit via the non-exclusive classification induced by the results of the directed circle Hough transform. The coordinates in the ridge container are clustered based on annuli masks dictated by the detected rings and sub-pixel accuracy of the rings parameters is achieved.

Additional notes

- The ridge detection can be used to achieve a compressed representation of the features in the image. This can be done by storing a hash table associating ridge coordinates as keys with their corresponding X_- as values.

- The algorithm is not restricted to directed ridges as it can be replaced by directed edges in case these are better descriptors of the features in the image. This is achieved by replacing the Hessian by the Gradient. In this case, the gradient magnitude replacing k_- has to be a local maximum along the gradient direction.

- To reduce false detection, the radii range is extended such that the Hough transform is over the

range $[r_{min} - 1, r_{max} + 1]$, but local maximum detection are searched for within the original range.

- In case additional performance per processing unit is required, one could use a lower resolution in discretising the circle parameter space. Measuring the effect of this on the accuracy is left for future work.
- Using several colours, the method should be, in principle, extendible to even higher particle densities.

Application of the proposed algorithm for particle tracking and discussion of alternative methods

When tracking small light emitting objects, such as fluorescent particles under the microscope, the appearance of rings is often a sign of the object going out of focus. Normally this results in the loss of the tracked object, which is thereafter considered as a hindering background source. However, these rings carry information of the 3-dimensional position of the particle. This has been used for localising a single light scattering magnetic bead based on matching the radial intensity profile to an empirical set of reference images [5]. An axial range of 10 µm was demonstrated and a temporal resolution of 25 Hz was achieved using the knowledge of the particle's previous position. In fact, for fluorescent particles the radius of the most visible ring of each particle precisely indicates its axial position – the radius follows a simple scaling with the particle distance from the focal plane (see Supplementary Fig. S1). A similar approach was recently described in [13], where the measurements were, once again, limited to a single particle in the observation volume, with an axial range of 3 µm and temporal resolution of 10 Hz.

In comparison with other existing methods for 3d particle tracking, the method presented here is advantageous when it comes to long measurements, temporal resolution and concurrency, as well as real-time applications. The confocal scanning microscope requires scanning the volume of interest. Therefore it is slower and cannot yet provide instantaneous information of the whole volume. Unlike Holographic microscopy [34, 35], the proposed method does not pose long and heavy computational demands which is restrictive for real-time applications or when large datasets are required for statistics.

One could expect the optical method discussed here to produce patterns which are symmetric about the focal plane. When this applies, it may result in an ambiguity with respect to whether the particle is above or below focus. Our optical arrangement (see the Methods section) shows clear diffraction rings only on one side. Furthermore, as particles approach focus, the radius of the outer-most ring becomes too small to resolve. For these reasons the focal plane is placed outside the volume of interest (as reflected in the Supplementary Fig. S1). Optical astigmatism offers a mean for discriminating between the two sides of the optical axis [36, 37, 38]. The introduction of a cylindrical lens results in the deformation of a circular spot into an ellipsoidal one as a fluorescent particle goes further away from focus, with the ellipse major axis of a particle above focus aligned perpendicular to a one below. In Ref. [36] the axial range was limited to a couple of microns above and below focus; in Refs. [37, 38] it was restricted to less than a micron. Within these ranges the tracer image can be approximated by an elliptical gaussian pattern. However, extending the range generates elliptical rings as well; see Figure 1 in Ref. [36]. This requires dealing with two species of patterns, spots and rings. Moreover, deforming circular rings into elliptical ones, the dimensionality of the parameter space increases, and so does the technical complexity of the image analysis. Therefore the advantage of the stronger signal, by working closer to focus on both its sides, is expected to have a heavy computational cost once the range is extended such that diffraction rings appear as well. The method presented here requires working away from focus. Rings visibility decreases as the fluorescence signal spreads over a larger area, thus setting the lower bound for the exposure time. Nevertheless, I have found that the fluorescence signal-to-noise ratio allowed tracking particles moving chaotically at speeds exceeding 400 µm/s.

Supplementary figures

a

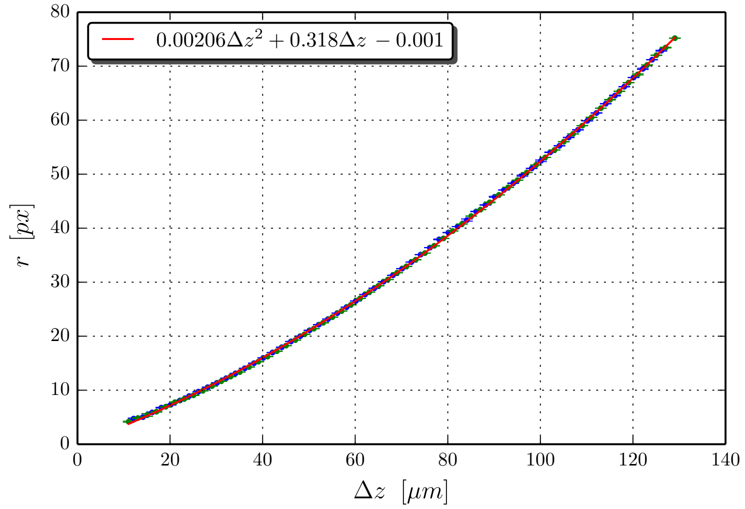

b

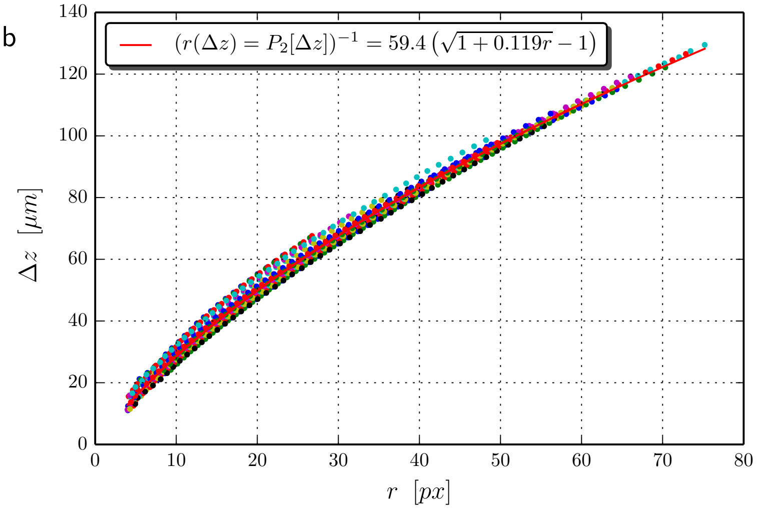

Supplementary Figure S1: **Calibration curve.** (**a**) The empirical relation between the outer-most ring radius and the out-of-focus distance Δz; the latter measures the translation of the objective from the position at which a particle would be in focus. The plot shows the data for two different fluorescent particles (denoted by blue and green in the plot). The quadratic polynomial fit provides an approximation for the $r(\Delta z)$ relation. The data was acquired by scanning through the vertical axis of the observation volume by objective translation steps of 2 µm (see Experimental details in the Methods section). Each data point is an average of the measured radius over 210 frames spanning 3 s, taken while the objective is stationary. Error bars reflect the standard-deviation; the median standard-deviation of the presented datasets is 0.03 px and the maximal is 0.27 px.

(**b**) The conversion function $r^{-1}(r)$ was obtained by the inversion of the quadratic polynomial fit, based on 25 tracers dispersed in the observation volume (see Experimental details in the Methods section). The resulting root-mean-squared-error $\sqrt{\langle(\Delta z - r^{-1}(r))^2\rangle} = 1.97$ µm, and the maximal measured absolute error is 5.35 µm; these estimate the uncertainty due to the calibration procedure followed here. Finally, the out-of-focus distance of the objective Δz needs to be converted to a physical distance via multiplication by the refractive indices ratio, 1.58 in this case. Thus the observed axial range exceeds 180 µm.

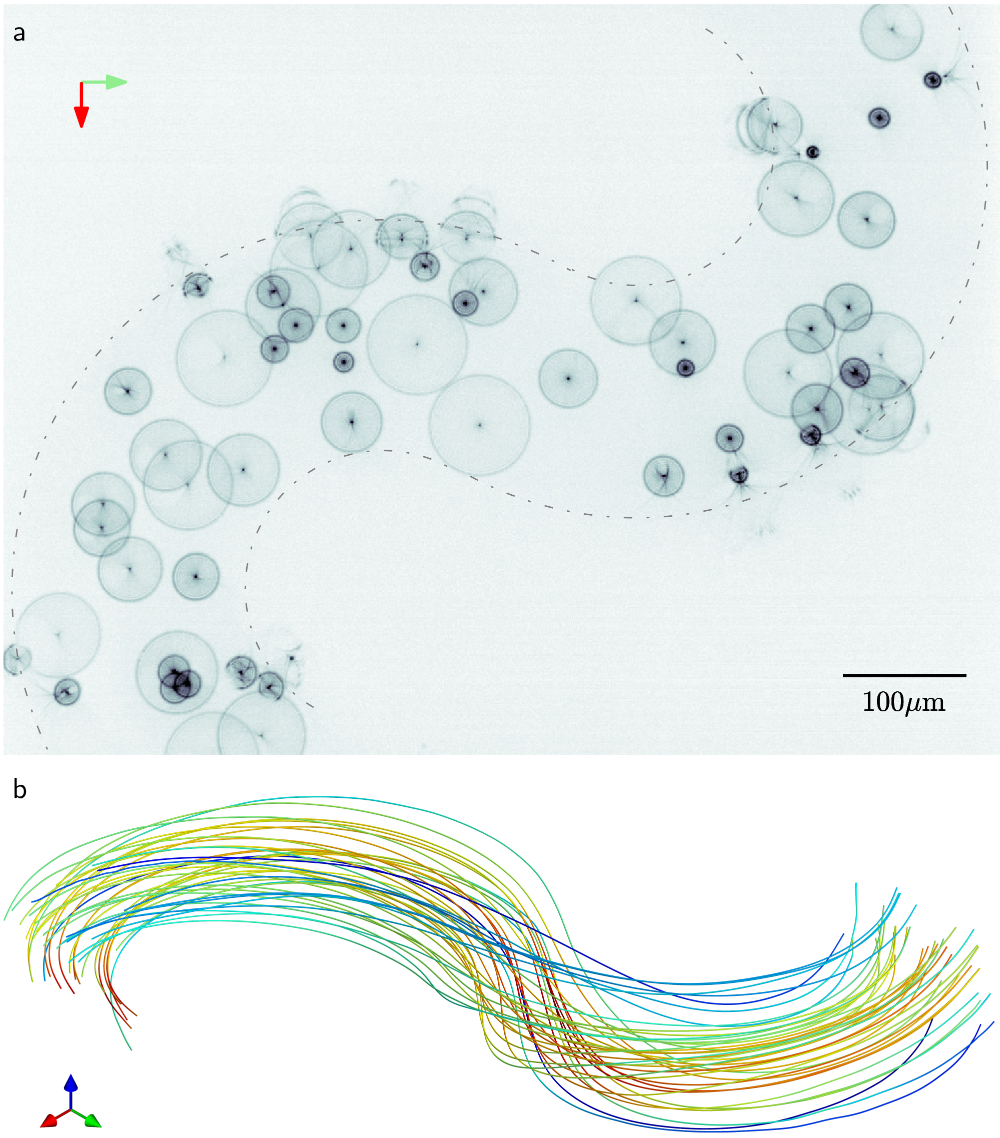

Supplementary Figure S2: **2d out-of-focus images → 3d trajectories.** (**a**) A single raw full 2d frame imaging a volume of 810 µm×610 µm×140 µm, including 60 tracers in one period of the curvilinear tube (which boundaries are denoted by the broken grey line). Smaller rings result from tracers being closer to the focal plane, which is positioned above the tube. (**b**) A sub-sample of 40 trajectories reconstructed from a time-lapse sequence of such frames, which spans 12 seconds of data acquisition. The colour coding indicates the speed ranging from 80 µm/s (blue) to 400 µm/s (red); the mean flow is rightwards. Corresponding axes are denoted by colours. The isometric view of the bottom panel can be obtained by three rotations, starting from the orientation of the upper panel: −90° about the green axis, −45° about the blue axis, and approximately 35° about the new horizontal axis.

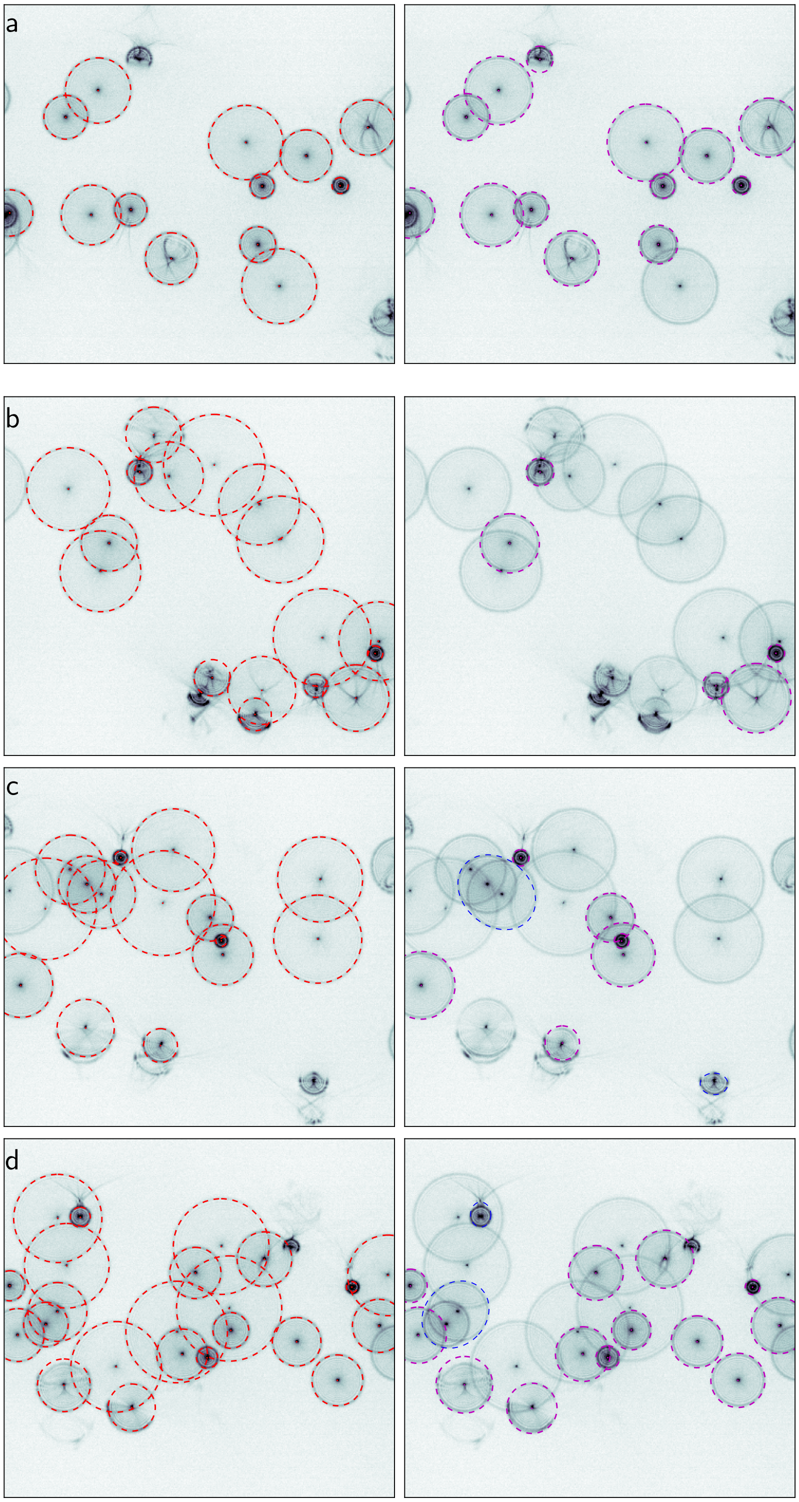

Supplementary Figure S3: **Examples from the comparative assessment of the algorithm robustness.** Output examples of the proposed algorithm can be found on the left column (reported rings are shown in dashed red) to be compared with those of ED-Circles [20] on the right column (circles in purple, ellipses in blue); further details of the test and results can be found in the Methods section. In 3.3% of the 151 tested images the alternative algorithm showed comparable results to those of the new one, that is both exhibited the same detection and error rates – an example is shown in (**a**); in all other examined images the proposed method outperformed its competitor – examples are shown in (**b**), (**c**) & (**d**). The new algorithm resolves rings even when the signal-to-noise ratio is limiting for the opponent – this is typical for tracers which are farther out-of-focus, hence their rings are larger and fainter. Overlapping rings of similar radii are another challenge resolved by the new algorithm; in contrast these are often missed or merged into ellipses by the opponent – see (**c**) & (**d**); similarly, optical artefacts often result in errors for the alternative method, in contrast to the new proposed one – e.g. see small ring reported by the opponent to be an ellipse in (**d**).
